# Variability of pMGA/vlhA sequences among Mycoplasma gallisepticum field strains isolated from laying hens and their deformed eggs

**DOI:** 10.1099/acmi.0.000681.v5

**Published:** 2024-06-13

**Authors:** Linda M. Maya-Rodríguez, Gabriela Gómez-Verduzco, Francisco J. Trigo-Tavera, Leticia Moreno-Fierros, Rosa E. Miranda-Morales

**Affiliations:** 1 Departamento de Microbiología e Inmunología, Facultad de Medicina Veterinaria y Zootecnia, Ciudad Universitaria, Universidad Nacional Autónoma de México, CDMX, 04510, México; 2 Departamento de Medicina y Zootecnia de Aves, Facultad de Medicina Veterinaria y Zootecnia, Ciudad Universitaria, Universidad Nacional Autónoma de México, CDMX, 04510, México; 3 Departamento de Patología, Facultad de Medicina Veterinaria y Zootecnia, Ciudad Universitaria, Universidad Nacional Autónoma de México, CDMX, 04510, México; 4 Facultad de Estudios Superiores Iztacala, Unidad de Biomedicina (UBIMED), Los Reyes Ixtacala, Universidad Nacional Autónoma de México, Tlanepantla de Baz, 54090, México

**Keywords:** avian, eggs, lipoprotein, mycoplasma, veterinary

## Abstract

Mycoplasmosis, attributed to *Mycoplasma gallisepticum*, poses a significant challenge to poultry farming, leading to substantial economic losses and persistent infections within flocks. This bacterium harbours various surface proteins that are crucial for adhesion, transporter activity and evasion of the host immune response, facilitating its pathogenicity. One such key surface lipoprotein, referred to as pMGA or vlhA haemagglutinin, plays a pivotal role in adhesion processes. In this study, the clonal regions pMGA1.2 and pMGA1.3, as reported by Markham (M83178.1), were investigated to elucidate differences or similarities in the whole DNA sequences of *M. gallisepticum* field strains. The aim was to analyse sequence diversity within this region. Six internal primers were designed to amplify the target sequence, and isolates were obtained from both eggs and chickens sourced from laying hen flocks. Identification revealed 17 strains of *M. gallisepticum* and four strains of *Mycoplasma synoviae*, which were confirmed through the *mgc2* and 16S rRNA genes, respectively. Positive and negative controls were established using the MGS6 and MSWUV1853 strains. Amplification results indicated a higher frequency of amplification proximal to the C-terminal region, with segments 4 (33.3 %) and 6 (27.8 %) being the most prevalent. Notably, none of the field strains exhibited the same amplification pattern as MGS6, and none of the strains characterized as *M. synoviae* amplified any primer set. Upon translation, the amino acid sequences from segments 4 and 6 were found to be compatible with conserved sequences within the Myco_haema protein domains of the genus *Mycoplasma*, specifically corresponding to Q7NAP3_MYCGA VlhA.3.04. The observed homology suggests a potential genetic transfer, while the variability identified in the *pMGA* or *vlhA* gene region of the field strains may have significant implications for protection against *M. gallisepticum* infection in chickens.

**Impact statement**

Significant variability in the sequences of *Mycoplasma gallisepticum* field strains compared to the reference sequence (M83178.1) was observed. Notably, only two segments of sequences were consistently amplified, while these segments were not amplified for the negative control, *Mycoplasma synoviae* and two field strains. The observed differences in the sequences could be attributed to the ineffective protection conferred via attenuated bacterial vaccines, which are designed to prevent disease manifestation in chickens.

## Data Summary

The following NCBI GenBank accession numbers were used: M83178.1 for primer design and L28424 for the comparison of sequences in the Discussion. Internal amplification sequences, as well as the amino-acid-translated sequences, are presented in the supplementary material with the online version of this article.

## Introduction

The bacterium *Mycoplasma gallisepticum* can be transmitted horizontally and vertically, leading to avian mycoplasmosis. This condition encompasses respiratory symptoms and infections in the oviduct, consequently affecting the eggs [1]. Despite lacking a cell wall, this bacterium possesses numerous surface proteins in its membrane. Some of these proteins are crucial for infection, including adhesins such as GapA and CrmA, fibronectin-binding proteins such as PlpA and Hlp3, a sugar transport protein called MalF [2], and the dihydrolipoamide dehydrogenase Lpd [3]. Additionally, lipoproteins with antigenic variability aid the bacterium in evading the host’s immune system. One such lipoprotein is haemagglutinin, referred to as vlhA [4]. In other studies, a lipoprotein known as pMGA was identified with amino acids resembling those of vlhA and the research concluded that are the same lipoprotein [5].

Previous work by Markham in the *M. gallisepticum* MGS6 strain showed that more than 50 genes encoded the lipoprotein pMGA, representing over 10 % of the genome [6]. The clonal sequence M83178.1, reported as pMGA 1.2 and pMGA 1.3, was studied, and the pMGA1.2 gene was identified as an important region for adhesion in a cell culture [3]. The receptor involved in pMGA cell interaction has been compared to ApoA-I, a high-density lipoprotein distributed in many chicken organs, with similarities to ApoE in mammals [3,7]. Interestingly, for this lipoprotein, amino acid similarities to the vlhA haemagglutinin, which is a sialic acid receptor, have also been reported [8]. The first report of pMGA indicated the presence of 30–70 genes related to transcription [8]. Mature pMGA contains a cysteine at the N-terminus, and its assembly is similar to the vlhA antigenic variability at the C-terminus [9,10]. The pMGA1.2 clonal gene from the MGS6 strain encodes a polypeptide variant that shares over 90 % similarity to vlhA [8]. Promoter regions identified as GAA have been reported in both vlhA and pMGA, supporting the notion that they represent the same gene [4,11]. The sequence M83178.1 has not been studied in field strains and since this sequence is reported to match with sequences of vlhA, it is not considered as specific for some mycoplasma species, although study of this sequence in field strains is convenient, since the haemagglutinin is involved in pathogenicity and antigenicity in vaccines against *M. gallisepticum* and *Mycoplasma synoviae*.

The aim of this study was to determine the similarities or differences of the pMGA 1.2 and pMGA 1.3 clonal sequences (M83178.1) in field strains of *M. gallisepticum* in order to assess this region’s variability [12,13]. A bioinformatics analysis of the amplified sequences was conducted, and whole DNA extraction from field strains infecting commercial laying hens was utilized since diverse sequences could be a determining factor in the pathogenicity and spread of mycoplasmosis.

## Methods

### Sample collection

Sampling for biosecurity purposes is difficult in production farms, so we decided to collect samples from laying hens considered to be of no value for commercial purposes in Mexico. Therefore, a total of 114 deformed eggs were collected from nine farms, and 10 hens from one of the nine farm flocks of laying hens. Deformed eggs have a dysfunctional air camara, and this condition is beneficial for mycoplasmas, which are microaerophilic bacteria [14,15]. The laying hen lineages present on farms without mycoplasma vaccination were Hy-line and Bovans White, typical lineages located in the central zone of Mexico (Mexico City, Jalisco). The 10 hens were killed to obtain air sacs and oviducts, according to the criteria of the Subcommittee for the Care and Use of Experimental Animals (SICUAE). These farms were selected for sample collection based on suspicions of mycoplasmosis. The objective was to isolate field strains of *Mycoplasma* spp*.* for experimental use, without considering a specific species of avian mycoplasma.

The mycoplasma field strains from the samples (eggs, air sacs and oviducts) were isolated accorded to Kleven’s [16] approach using Frey culture medium [17]. For each egg, the white, yolk and shell membrane were analysed with six internal primer sets, based on Markham’s reported sequences [5]. The air sacs and oviduct mucosa were each sampled with a sterile swab. These samples were incubated in a Fisher Scientific incubator for up to 30 days in order to obtain mycoplasma-like colonies. For discrimination between *Mycoplasma* spp. and *Acholeplasma* spp., belonging to the class *Mollicutes*, colony morphology, filterability through a 0.45 µm filter and digitonin tests (Biotechnology) were used [17].

### Growth of field and reference strains

To determine the strains’ viability and purity, *Mycoplasma* isolates were grown in 100 ml of Frey culture medium [16,17] for approximately 30 days. The obtained culture was then seeded in Frey solid medium (Merck KGaA; 1.46311.000) and blood agar (Merck KGaA; 70133) to guarantee strain purity.

For all molecular tests, the *M. gallisepticum* MGS6 strain (ATCC 15302) – which was isolated in 1960 by Edward and Kanarek from the brain of a turkey with torticollis – was used as a positive control. The *M. synoviae* MSWVU1853 strain (ATCC 10124), isolated from the hock joint of a chicken, was used as a negative control.

### DNA isolation

The bacterial culture was centrifuged at 300 *g* for 45 min at 4 °C (Thermo Sorvall Legend RT Plus Refrigerated Benchtop Centrifuge). The pellet was washed three times with PBS (pH 7.2) [18]. DNA was extracted using guanidine thiocyanate (Biotechnology), following the methodology of Sambrook and Russell [18]. The isolated DNA was used for molecular typing with the endpoint PCR technique using MyTaq polymerase (Bioline, SKU: BIO-2110X).

### Identified primers

The following primers were used to amplify a coding fragment in order to identify *M. gallisepticum* and *M. synoviae* (Table 1). The thermal cycling protocol entailed denaturation at 95 °C for 4 min, 30 cycles of hybridization at 94 °C for 30 s, annealing at 55 °C for 16S RNA and 57 °C for *mgc2* for 1 min, extension at 72 °C for 1 min, and final extension at 72 °C for 5 min (Techne 3 Prime, Cole-Palmer Instrument Company). The thermal cycling conditions were based on previously applied protocols [19], but the annealing temperature was adapted, depending on the strain type (*M. gallisepticum* and *M. synoviae*) and the annealing temperature of the primers.

**Table 1.** Primers used for the identification of *M. gallisepticum, M. synoviae* and the six M83178.1 internal sequences

Primer name	Primer sequence (5′→3′)	Location	Product length (bp)
Rv *mgc2* MG	CGCAATTTGGTCCTAATCCCCAACA	825–847	
Fw *mgc2* MG	TAAACCCACCTCCAGCTTTATTTCC	498–518	349
Rv 6 pMGA	GATTTTCTAAAGTGGCGCTTG	2 789–2 800	
Fw 6 pMGA	GAAGTTCTTAGGAGTTCTGG	2 278–2 298	532
Rv 5 pMGA	CCAGAACTCCTAAGAACTTC	2 278–2 288	
Fw 5 pMGA	GACACCTCTGCAACAAC	1 855–1 871	434
Rv 4 pMGA	GTTGTTGCAGAGGTGTC	1 855–1 871	
Fw 4 pMGA	GATATTTTCGGTAATAGTGTTACAAC	1 429–1 445	452
Rv 3 pMGA	GTTGTAACACTATTACCGAAAATATC	1 429–1 445	
Fw 3 pMGA	CAGTGAACCTAGAAG	990–1 008	465
Rv 2 pMGA	CTTCTAGGTTCATCACTG	990–1 008	
Fw 2 pMGA	GTAAAAGTTTATAAAGAATTAAAAACGAC	544–572	455
Rv 1 pMGA	GTCGTTTTTAATTCTTTATAAACTTTTAC	544–572	
Fw 1 pMGA	CATATTAAAGTTTGTTAGTTTATTAGGTA	123–151	450
Fw*16S RNAr MS*	CAGTCGTCTCCGAAGTTAACAA	667 270–667 292	
Rv *16S RNAr MS*	CACAAGCGGTGGAGCATG	667 762–667 780	514

### Amplification of the internal segment of M83178.1 sequences in the *Mycoplasma* field strains

The variable region of lipoprotein was amplified with six internal primers, based on GenBank accession number M83178.1, ‘*Mycoplasma gallisepticum* haemagglutinin (pMGA1.2 and pMGA1.3) homologous genes, complete cds’ To ensure good amplification and sequencing results, it was necessary to divide the variable region into six internal segments, each <600 bp in length. Table 1 gives details of the primers. The thermal cycling conditions comprised denaturation at 95 °C for 4 min, 36 cycles of hybridization at 94 °C for 30 s, annealing at 50 °C for 1 min, extension at 72 °C for 1 min and final extension at 72 °C for 5 min. The DNA bands were visualized using a Gel Logic 212 Pro photo documenter (Carestream), and a lane with 1 kb DNA ladder (Invitrogen, Fisher Scientific Waltham) served as a molecular weight marker.

The PCRs that yielded visible amplicons were subjected to cleaning and sequencing at the Institute of Biology of Universidad Nacional Autónoma de México. The resulting sequences were then utilized to reconstruct a phylogenetic tree, which was analysed using BioEdit and mega x [20]. Additionally, conserved regions within the lipoprotein haemagglutinin of mycoplasmas were identified based on GenBank data using the muscle 3.8 software [21,22], https://bio.tools/muscle. These conserved regions were crucial for understanding the genetic similarities and differences among the studied strains and elucidating their evolutionary relationships.

## Results

A total of 17 *M. gallisepticum* strains, identified based on the *mgc2* gene, were isolated from the clinical samples obtained via commercial poultry farms. These strains were recovered from various sources, including eggs (15 strains: nine from the shell membrane, three from the yolk and three from the egg white), one strain from an oviduct mucosal sample and one strain from a left abdominal air sac. Additionally, four field strains of *M. synoviae*, typified by the 16S rRNA gene (NZ_CP021129.1 *M. synoviae* strain MS-H chromosome, complete genome), were recovered from three of the nine farms tested. Among these, two randomly selected *M. synoviae* strains were subjected to PCR testing for M83178.1 sequences.

PCR amplification of the M83178.1 sequences was successful for all the tested *M. gallisepticum* field strains. The PCR protocol provided a standardized result with 3 ng µl^–1^ of bacterial DNA which was necessary for amplification with 10 µl of DNA utilized at a final volume of 25 µl for each reaction. The segments most frequently amplified were segments 4, 5 and 6, which are located near the C-terminus of the protein. To confirm the PCR result with the negative control (MSWVU1853 strain), two field strains of *M. synoviae* (Y277 and F63) were tested. Neither of these strains exhibited amplification with any primer set (Table 2).

**Table 2.** Amplification of six segments of the variable region of the lipoprotein in *M. gallisepticum* field strains and the MGS6 strain

Strain ID	Segment 1	Segment 2	Segment 3	Segment 4	Segment 5	Segment 6	Sample	Farm
F3	−	−	−	−	✔	−	Shell membranes	1
C12	−	−	−	−	✔	−	White	1
F32	−	−	−	−	−	✔	Shell membranes	2
F40	−	−	−	−	✔	−	Shell membranes	2
Y41	−	−	−	✔	✔	−	Yolk	2
F42	−	−	−	−	−	✔	Shell membranes	2
F62	−	−	−	✔	✔	−	Shell membranes	3
C91	−	−	−	−	✔	−	White	4
Y132	−	−	−	−	✔	−	Yolk	5
F132	−	−	−	−	✔	−	Shell membranes	5
F162	−	−	−	✔	−	−	Shell membranes	6
F231	−	✔	✔	−	✔	−	White	9
C251	−	✔	−	−	−	✔	White	9
F281	−	−	−	−	✔	−	Shell membranes	9
Y252	−	−	−	−	✔	−	Yolk	9
B2M	−	−	−	✔	✔	−	Oviduct	9
B4SA	−	−	−	✔	−	✔	Air sacs	9
Y277	−	−	−	−	−	−	Shell membranes	3
F63	−	−	−	−	−	−	Shell membranes	9
MGS6	−	−	−	✔	✔	✔	ATCC	
MSWUV1853	−	−	−	−	−	−	ATCC	

The sequences obtained from the blast online software yielded identities of 95.20–90 %, with 38 % coverage specifically matching *M. gallisepticum* strains as avian mycoplasmas. However, there was lower identity and coverage observed with other bacteria containing haemagglutinin in segment 4. Regarding segment 6, the identity ranged from 96.6 to 89.6 %, with coverage of 51–35 %. Similar to segment 4, the matches were primarily with *M. gallisepticum* strains, with lower identity and coverage observed with other bacteria.

Segment 1 was not amplified in any of the tested strains. Meanwhile, segment 2 was observed to have been amplified in only one strain (F231), and segment 3 was observed to have been amplified in two strains (F231 and C251). Notably, the MGS6 strain did not exhibit amplification of segments 1–3 although *in silico* analysis indicated a match. However, differences between the MGS6 strain and the field strains were observed; more details are given in the Discussion.

**Fig. 1.**
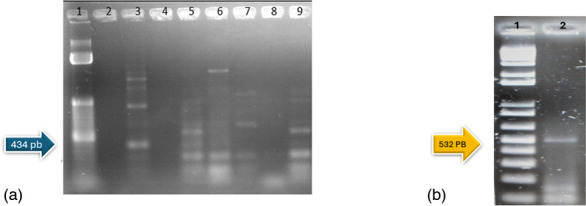
(a) Gel electrophoresis of the PCR detection for segment 5 of the pMGA1.2 and pMGA1.3 variable regions. Lane 1, 1 kb DNA ladder (Invitrogen); lane 2, F162 (negative); lane 3, F231 (positive); lane 4, C251 (negative); lane 5, F281 (positive); lane 6, Y252 (positive); lane 7, B2M (positive); lane 8, B4SA (negative); and lane 9, MGS6 (positive). (b) Gel electrophoresis of the PCR detection of segment 6 of the pMGA1.2 and pMGA1.3 variable regions. Lane 1, 1 kb DNA ladder (Invitrogen); lane 2, MGS6 (positive).

A bioinformatics analysis of the nucleotide sequences obtained via amplification was performed only for fragments 4 and 6. This decision was made because nonspecific amplification was observed in fragment 5, even in the MGS6 strain, as shown by the electrophoresis results (Fig. 1a). Therefore, fragments 4 and 6 were selected for further analysis due to their specificity (Fig. 1b) and because they were the most commonly observed segments.

In segment 6, multiple alignments revealed fewer than 20 mutations due to transversion and three mutations due to transition. Similarly, in segment 4, there were 19 mutations due to transversion and 13 mutations due to transition. Notably, both sequences exhibited continuous and discontinuous sequences of GAA in tandem.

**Fig. 2.**
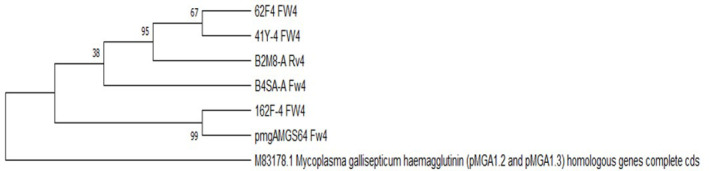
Phylogenetic tree of segment 4 of *pMGA1.2* and *pMGA1.3* amplified in four field strains and the MSG6 strain, as well as the M83178.1 sequence. The evolutionary history was inferred, and the percentage of associated taxa clustered together is shown next to the branches. The final data set included a total of 2050 positions. Evolutionary analyses were conducted using mega x.

**Fig. 3.**
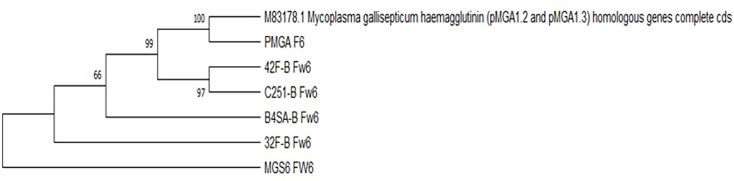
Phylogenetic tree of segment 6 of *pMGA1.2* and *pMGA1.3* amplified in five field strains and the MSG6 strain, as well as the M83178.1 sequence. The evolutionary history was inferred, and the percentage of trees in which the associated taxa clustered together is shown next to the branches. The final data set included a total of 2815 positions. Evolutionary analyses were conducted in mega x.

Based on the results of the bioinformatics analysis, phylogenetic trees were reconstructed using the maximum-likelihood method and the Tamura–Nei model [22]. In segment 4, a cluster relationship was observed among the five field strains that were amplified, including three strains from eggs, one strain from an air sac and one strain from an oviduct. A bootstrap consensus tree indicated a high degree of similarity among the sequences (Fig. 2). Similarly, in segment 6, a clustering relationship was observed among the four field strains, including three strains from eggs and one strain from an air sac, with a significant level of similarity (Fig. 3).

### Translation of the sequences to amino acids

**Fig. 4.**
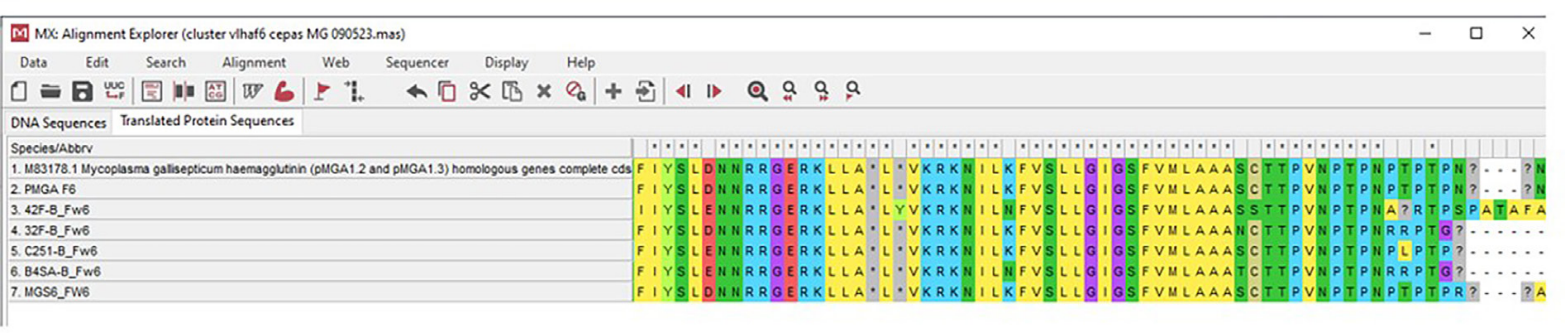
Alignment via mega x software revealed that, of the strains that amplified segment 6 (four field strains – 42F, C251, B4SA and 32F – and MGS6), many amino acids aligned with Q7NAP3.

Multiple-sequence alignment was performed via muscle 3.8 for segments 4 and 6 (https://www.ebi.ac.uk/Tools/msa/muscle/), showing a conserved protein with the blast tool of NCBI for the domain family Myco_haema in alignment with the Q7NAP3 sequence, which encodes vlhA 3.04 haemagglutinin in *M. gallisepticum* (Supplementary Material, available in the online version of this article). In segment 6, clustal muscle multiple-sequence alignment showed that many amino acids aligned with the Q7NAP3 sequence (Fig. 4).

## Discussion

The 17 *M. gallisepticum* strains isolated from eggs (6.3 %) and from oviducts and air sacs (10 %) correspond with the reported prevalence of avian mycoplasmas in poultry, which ranges from 7 to 23 % [23,24]. Since the M83178.1 model sequence represents a variable region, amplifying all internal segments (corresponding to 2 803 bp) in field strains was impossible due to the unknown status of the sequence and the type of *M. gallisepticum* strain. However, amplification was achieved in different internal segments. The MGS6 ATCC positive control only exhibited amplification of segments 4–6, indicating a specific amplification pattern. Cluster analysis also revealed an amino acid grouping shared with that of other studies, consistent with a 71 % similarity in a consensus domain among bacterial genera [25,26]. These results suggest variation close to the C-terminal regions in the lipoproteins, consistent with previous reports that have indicated their recombinant nature [3,27]

An *in silico* analysis was conducted using the complete genome of the MGS6 strain (GenBank NC_023030.2) and the L28424 GenBank sequence, corresponding to haemagglutinin homologues with M83178.1, which served for the primer design. Primers directed toward segments 1, 2 and 3 of the internal sequences presented deletions at nucleotide positions 1 201, 1 228, 1 239, 1 240 and 1 251 in the FASTA sequences and at nucleotide positions 121 and 541 in the reference sequence. This variability in sequences of internal primers 1 and 2 may underlie the different amplification patterns observed in the field strains, explaining the variability identified in the protein. Primer set 5 matched a different region in the whole genome of * M. gallisepticum* sequences, which could explain the results obtained in this study, indicating an unspecified match. Although 17 field strains and the ATCC strain of *M. gallisepticum* were tested using DNA extraction in the PCR, variability in pMGA or vlhA – which are composed of a group of genes – may have contributed to the observed variations. However, fragments 4 and 6 were only amplified in *M. gallisepticum* and not in *M. synoviae*, WVU1853, or the two field strains Y277 and F63 even though both strains had the same genes and the same conditions were employed.

The results of the analysis of sequences from regions 4 and 6 are more compatible with those reported by other authors [25], reaffirming the genetic similarity present in the vlhA gene and showing the same promoter sequence, GAA [28]. On the other hand, in an unpublished study with reported vlhA sequences (AF035624.1) using the same strain employed in the current study, amplification could be observed in both *M. synoviae* and *M. gallisepticum* when visualizing only one amplicon and utilizing whole DNA extraction. Since Markham determined that the vlhA protein shares 41 % of its amino acid sequences with the pMGA 1.1 region sequence, which closely resembles vlhA 1 [3], the expected result regarding the sequence of * M. synoviae* strains matches with the primers used, since vlhA and pMGA are from the same family. However, this matching did not occur, which indicates that more studies of different *M. synoviae* strains are needed.

The variability identified in lipoproteins can be influenced by behaviour and genomic sequences that often lead to antigenic variability. This phenomenon occurs in vlhA, and it has been reported in other proteins, such as Omp-PA [29]. Variability helps in the infection process and with gene expression, depending on the stage of infection with *M. gallisepticum* [27]; although the infection status of the strains used in the current study was unknown, the results still demonstrate variation in field strains, as reported.

Chronic *M. gallisepticum* infection leads to variability in lipoprotein expression among different cultured cells [8]. The on or off status of genes is influenced by the host’s behaviour and the bacterium’s metabolic needs. With the pMGA or vlhA gene, gene regulation is based on switches to a stable alternative phase after a cell is invaded by modulating the C-terminal region. The lipoprotein antigenic mechanisms involve masking and unmasking processes. These changes modulate antibody binding to conserved sites on surface proteins, allowing cross-antigenic reactions among species with the same or similar proteins [30]. In bacterial families or groups with similar characteristics, finding regions with surface-protein similarities is not difficult.

The diversity in the nucleotide sequences found in this study among field strains of *M. gallisepticum* influences the efficiency of vaccines that employ attenuated strains, such as strains MG F, t-11 and 6/85 [31,33]. In mycoplasma infections, lipoproteins are very important for adhesion, colonization and immune system evasion [34,35].

## Conclusions

The pMGA1.2 and pMGA1.3 (M83178.1) clonal sequences analysed in this study revealed sequence diversity among * M. gallisepticum* field strains, which could contribute to their immune evasion capacity and adaptability. The amino acid sequence similarity to QA9487 was confirmed in field strains. However, the strain typified as *M. synoviae* did not amplify any region of the studied sequence. This finding presents an opportunity to study the variability in the lipoprotein of this bacterium, which is relevant to infection.

## supplementary material

10.1099/acmi.0.000681.v5 Uncited Supplementary Material 1.

## Abbreviations

ApoA: apolipoprotein A
ApoE: apolipoprotein E
ATCC: american type culture collection
M. gallisepticum: Mycoplasma gallisepticum
M. synoviae: Mycoplasma synoviae
Myc: Mycoplasma
PBS: phosphate-buffered saline
PCR: polymerase chain reaction
pH: potential hidroxin
Tm: alienation temperature
USA: United Stated of America
