# Supplementary material for: Variability of pMGA/vlhA sequences among Mycoplasma gallisepticum field strains isolated from laying hens and their deformed eggs
Source: Access Microbiol. 2024 Jun 13;6(6):000681.v5. doi: 10.1099/acmi.0.000681.v5 (PMC11261720; doi:10.1099/acmi.0.000681.v5)

Amino acid multiple sequences from fragment 6 of pMGA.

```
CLUSTAL multiple sequence alignment by MUSCLE (3.8)


sp|Q7NAP3|Q7NAP3_MYCGA      MKRKNILKFVSLLGIGSFVMLAAASCTTPVNPTPNPTPNPTPTPNPEPNPPSGGNMNGGN
B4SA-B_Fw6                  ----------------------WIWNRSR-------------------------------
32F                         --------------------KQMAGLRQP-------------------------------
MGS6_FW6                    --------------------AGLSGYRIP-------------------------------
M83178.1                    ------------------------------------------------------------
C251-B_Fw6                  --------------------KELXVCRXP-------------------------------
42F-B_Fw6                   --------------------XKLIICRRR-------------------------------


sp|Q7NAP3|Q7NAP3_MYCGA      TNPSDGQGMMNAAAKELADAKAALTSLIGSKDQNTKLYADYEKIQDTLVKAYDAAEATLN
B4SA-B_Fw6                  -------HLSSPLXXKKKKKKKKFLGVLG-------------------------------
32F                         -------GLWSALTTKKKKKKKKFLGVLG-------------------------------
MGS6_FW6                    -------GXWSSLXQKKKKKKKKFLGVLG-------------------------------
M83178.1                    ------------XKKKKKKKKKKFLGVLG-------------------------------
C251-B_Fw6                  -------GXCPSRXXKKKKKKKKFLGVLG-------------------------------
42F-B_Fw6                   -------GXWPSRXXKKKKKKKKFLGVLG-------------------------------
                                           :  . *  :  .::*

sp|Q7NAP3|Q7NAP3_MYCGA      NSASTTQNLKDAETTLQAAISTAASSKQTFNQQNNALVTAYNQLKTAVGNESATLAKVED
B4SA-B_Fw6                  ------------------------------------------------------------
32F                         ------------------------------------------------------------
MGS6_FW6                    ------------------------------------------------------------
M83178.1                    ------------------------------------------------------------
C251-B_Fw6                  ------------------------------------------------------------
42F-B_Fw6                   ------------------------------------------------------------


sp|Q7NAP3|Q7NAP3_MYCGA      AKFARIKENLVTLYNAGKMLTAKPLEGIDGAVLDVDQVTQANTKLQEATNENSLTQQTQN
B4SA-B_Fw6                  -------------FRAGLI-----------------------------------------
32F                         -------------FRGVLM-----------------------------------------
MGS6_FW6                    -------------FRAGLF-----------------------------------------
M83178.1                    -------------FRAGLI-----------------------------------------
C251-B_Fw6                  -------------FRAGLI-----------------------------------------
42F-B_Fw6                   -------------FRAGLI-----------------------------------------
                                         :..  :

sp|Q7NAP3|Q7NAP3_MYCGA      ATNLADSFVKQVINKTKITGTTNTEKQPGNYSFVGYSVDLNTITTRTGNNSQTSSSSDNL
B4SA-B_Fw6                  -----------------------------------------------GGNKR-----DLL
32F                         -----------------------------------------------GENKR-----DLL
MGS6_FW6                    -----------------------------------------------RENKR-----DLL
M83178.1                    -----------------------------------------------GENKR-----DLF
C251-B_Fw6                  -----------------------------------------------GENKR-----DLF
42F-B_Fw6                   -----------------------------------------------GENKR-----DLF
                                                                             *..     * :

sp|Q7NAP3|Q7NAP3_MYCGA      PSWNFAQRIVWTNTANKNFPLADQTENSVPLTDVSWIYSLSGMNAKYTLTFNYYGPDTAY
B4SA-B_Fw6                  GNWTL--YILXR------------------IIDV--------VNVSYWLNFKW-------
32F                         DNWTL--YILXT------------------IIDV--------VNVSYWLNFKW-------
MGS6_FW6                    DNWTL--YILXT------------------IIDV--------VNVSYWLSFKW-------
M83178.1                    HNWTL--YILXT------------------IIDV--------VNVSYWLNFKW-------
C251-B_Fw6                  HNWSL--YILXR------------------IIDV--------VNVSYWLNFKW-------
42F-B_Fw6                   HNWTL--YILXR------------------IIDV--------VNVSYWLNFMW-------
                             .*.:   *:                    : **        :*..* *.* :
```

```
sp|Q7NAP3|Q7NAP3_MYCGA      LYFPYKLVKTNDRAELQYSLNGANAKLIEFKPAPTVPPVQPPAAPAPAERAATETAANSN
B4SA-B_Fw6                  ------------------------------------------------------------
32F                         ------------------------------------------------------------
MGS6_FW6                    ------------------------------------------------------------
M83178.1                    ------------------------------------------------------------
C251-B_Fw6                  ------------------------------------------------------------
42F-B_Fw6                   ------------------------------------------------------------

sp|Q7NAP3|Q7NAP3_MYCGA      QTNQEGSDTNQAMPATATMNEAPTVADINVAKVKLTGLKFGENTIDFSVPQSTGSDMSKV
B4SA-B_Fw6                  ------------------------------------------------------------
32F                         ------------------------------------------------------------
MGS6_FW6                    ------------------------------------------------------------
M83178.1                    ------------------------------------------------------------
C251-B_Fw6                  ------------------------------------------------------------
42F-B_Fw6                   ------------------------------------------------------------

sp|Q7NAP3|Q7NAP3_MYCGA      APMIGNMYLTSSDTDANKNKIYDDLFGNTLVKQNNESTVTVDLLKGYSLATSYFEYIRQF
B4SA-B_Fw6                  -----------------KEKTFXTLLVYXVLVR----------LXCWQLLLVLHRSTRHL
32F                         -----------------KEKTFXSLLVYXVLVR----------LXCWQLLIVLHRSTQHQ
MGS6_FW6                    -----------------KEKTFXSLLVYXVLVR----------LXCWQLLVVLHRSTRHL
M83178.1                    -----------------KEKTFXSLLVYXVLVR----------LXCWQLLVVLHRSTQHL
C251-B_Fw6                  -----------------KEKTFXSLLVYXVLVR----------LXCWQLLVVLHRSTRHL
42F-B_Fw6                   -----------------KEKTFXTLLVYXVLVR----------LXCWQLLVLLHRSTQHQ
                                             *:* :  *:   :: .          *  :.*    .   .:

sp|Q7NAP3|Q7NAP3_MYCGA      TDLTAEGSATKSPTVYLVGLINGSARRTDAENVPTTPTSPNLTGNSRTFTIYVNAPQDGD
B4SA-B_Fw6                  TEGPLE--------ASRLII------------WPXAAAXRXARTRLRPR-----------
32F                         TEGRLE--------ARRLIA------------RPXAKAXRXARSRLRSR-----------
MGS6_FW6                    TQHPLQGAXPWASRARRLIA------------RPXAKAXRXARSRLRSR-----------
M83178.1                    TQHQLQT-------LNQILV------------VXXEVCLMEIPIQXEWW-----------
C251-B_Fw6                  TRFPLQS-------VXATVX------------RPTRPCWGSGRLXRLQHAL---------
42F-B_Fw6                   TXVGLQAR------XHRLRL------------VDRDRDLR-XRCRSXTYT----------
                            *    :

sp|Q7NAP3|Q7NAP3_MYCGA      YYISGSYLNGDNTSRQPTAKRYLKFSTTQGTEPNMNSSLVIDVKSLNSWTILGTFDTKTN
B4SA-B_Fw6                  ------RFNGSKMRS-RXDGRY-----------------------ADAIVIHPDF-----
32F                         ------RXNGSKMRS-RXVSRY-----------------------ADAIVVHPDF-----
MGS6_FW6                    ------RXNGSKMRS-RXVSRY-----------------------ADAIVVHPDF-----
M83178.1                    ------RYDGRQSXSWEHHTRT-----------------------TIXQLLEKRX-----
C251-B_Fw6                  ------RLDGRXMPMPRTSRTV-----------------------VMPWVLXRPP-----
42F-B_Fw6                   ------ILAGLQTVXRPTPSK------------------------PRRWTXRRRD-----
                                     *

sp|Q7NAP3|Q7NAP3_MYCGA      KNINEQTTSTGSGREGSSAQSANKTLHLKKGLNKVIIGGDKDLNTPYIGKLSFTLKISAD
B4SA-B_Fw6                  -------SSFGAYPHAHXGGSCX-------------------------------------
32F                         -------TAFGAYAHAHXGCACX-------------------------------------
MGS6_FW6                    -------TAFGAYAHM--------------------------------------------
M83178.1                    -------PTYLXYRKYQCCLVCWLCQNSKYFKHCLXWXAKTASENTXXPLXKIX------
C251-B_Fw6                  -----PXDAFVAHQRLLT------------------------------------------
42F-B_Fw6                   -------PHLLDGRRAPAAXAS--------------------------------------

sp|Q7NAP3|Q7NAP3_MYCGA      GSSSTPENVSGAAA
B4SA-B_Fw6                  --------------
32F                         --------------
MGS6_FW6                    --------------
M83178.1                    --------------
C251-B_Fw6                  --------------
42F-B_Fw6                   --------------
```

Cluster sequences of amplification from fragment 6 pMGA (M83178.1) in field strains.

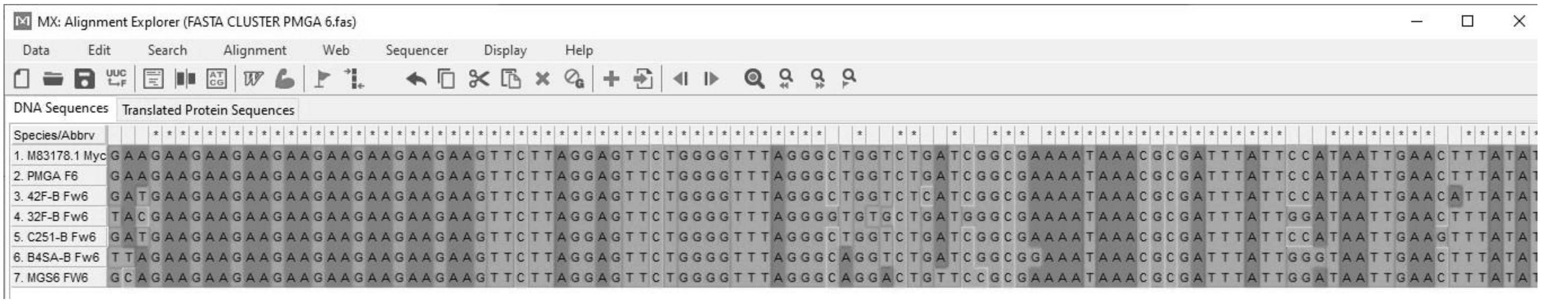

Cluster sequences of amplification from fragment 4 pMGA (M83178.1) in field strains.

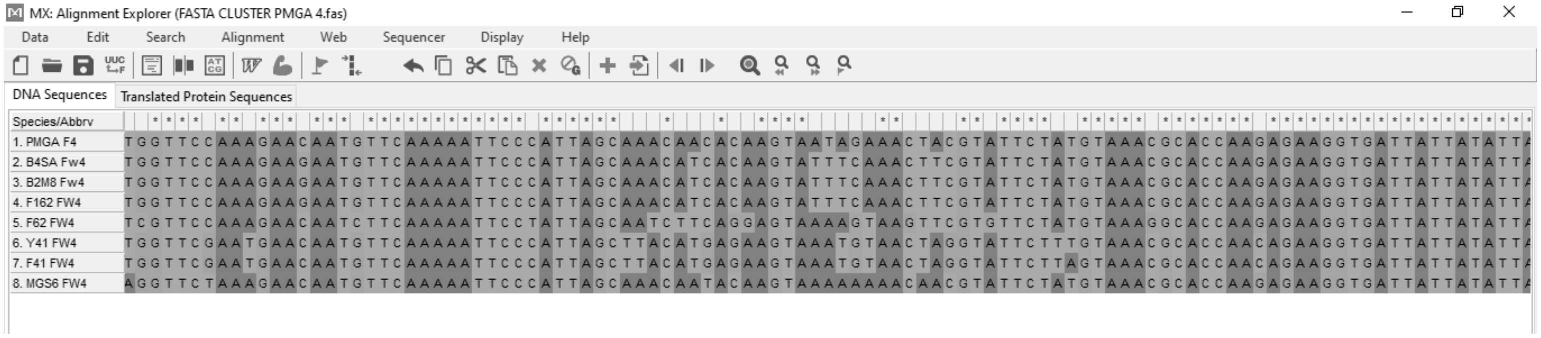

Supplement: Uncited Supplementary Material 1. [file acmi-6-00681-s001.pdf]
